# The Emerging Roles of Early Protein Folding Events in the Secretory Pathway in the Development of Neurodegenerative Maladies

**DOI:** 10.3389/fnins.2017.00048

**Published:** 2017-02-07

**Authors:** Tatyana Dubnikov, Ehud Cohen

**Affiliations:** Department of Biochemistry and Molecular Biology, The Institute for Medical Research Israel—Canada, The Hebrew University School of MedicineJerusalem, Israel

**Keywords:** chaperone, aggregation, endoplasmic reticulum stress, neurodegeneration, aggresome

## Abstract

Although, protein aggregation and deposition are unifying features of various neurodegenerative disorders, recent studies indicate that different mechanisms can lead to the development of the same malady. Among these, failure in early protein folding and maturation emerge as key mechanistic events that lead to the manifestation of a myriad of illnesses including Alzheimer's disease and prion disorders. Here we delineate the cascade of maturation steps that nascent polypeptides undergo in the secretory pathway to become functional proteins, and the chaperones that supervise and assist this process, focusing on the subgroup of proline *cis/trans* isomerases. We also describe the chaperones whose failure was found to be an underlying event that initiates the run-up toward neurodegeneration as well as chaperones whose activity impairs protein homeostasis (proteostasis) and thus, promotes the manifestation of these maladies. Finally, we discuss the roles of aggregate deposition sites in the cellular attempt to maintain proteostasis and point at potential targets for therapeutic interventions.

The generation of a polypeptide by the ribosome is the first step of the long and complex process that leads to the formation of a mature, functional protein. Cytosolic proteins maturate at the cytosol (Hartl and Hayer-Hartl, 2002) while secreted and membrane proteins are processed at the secretory pathway (Ellgaard and Helenius, 2003). Here we focus on protein folding and maturation within the secretory pathway and delineate how failures in this process underlie the manifestation of certain cases of late-onset neurodegenerative disorders.

## Protein maturation and quality control within the endoplasmic reticulum

The first domain of many nascent chains of a secretory proteins to exit the ribosome is a hydrophobic signal sequence of 20–30 amino acids that targets the polypeptide into the endoplasmic reticulum (ER; Hegde and Bernstein, 2006). The appearance of the signal peptide, and its recognition by the signal recognition particle (SRP; Lauffer et al., 1985), leads to the binding of the translating ribosome to the ER channel protein complex Sec61p (Sanders et al., 1992) and to the co-translational insertion of the nascent polypeptide into the ER lumen. This translocation mechanism is not exclusive as newly synthesized polypeptides can enter the ER lumen through alternative mechanisms that have been discovered in recent years. A subgroup of “tail-anchored (TA) proteins” bear a C-terminal hydrophobic trans-membrane domain that interacts with the family of GET proteins (guided entry of TA proteins). In yeast, the TA protein-GET interactions promote the post-translational entry into the ER by a SRP-independent mechanism (Schuldiner et al., 2008). Mammalian TA proteins enter the ER through an analogous mechanism that requires the GET orthologue Asna1/TRC40. Interestingly, under certain circumstances, the TRC40-dependent ER translocation mechanism cooperates with the canonical Sec61p apparatus to orchestrate proper post-translational entry of polypeptides into the ER (Johnson et al., 2012).

An additional ER translocation mechanism that functions independently of SRP and GET proteins but depends upon the activity of the Snd1, Snd2 and Snd3 proteins has been discovered recently (Aviram et al., 2016).

**Figure 1:**
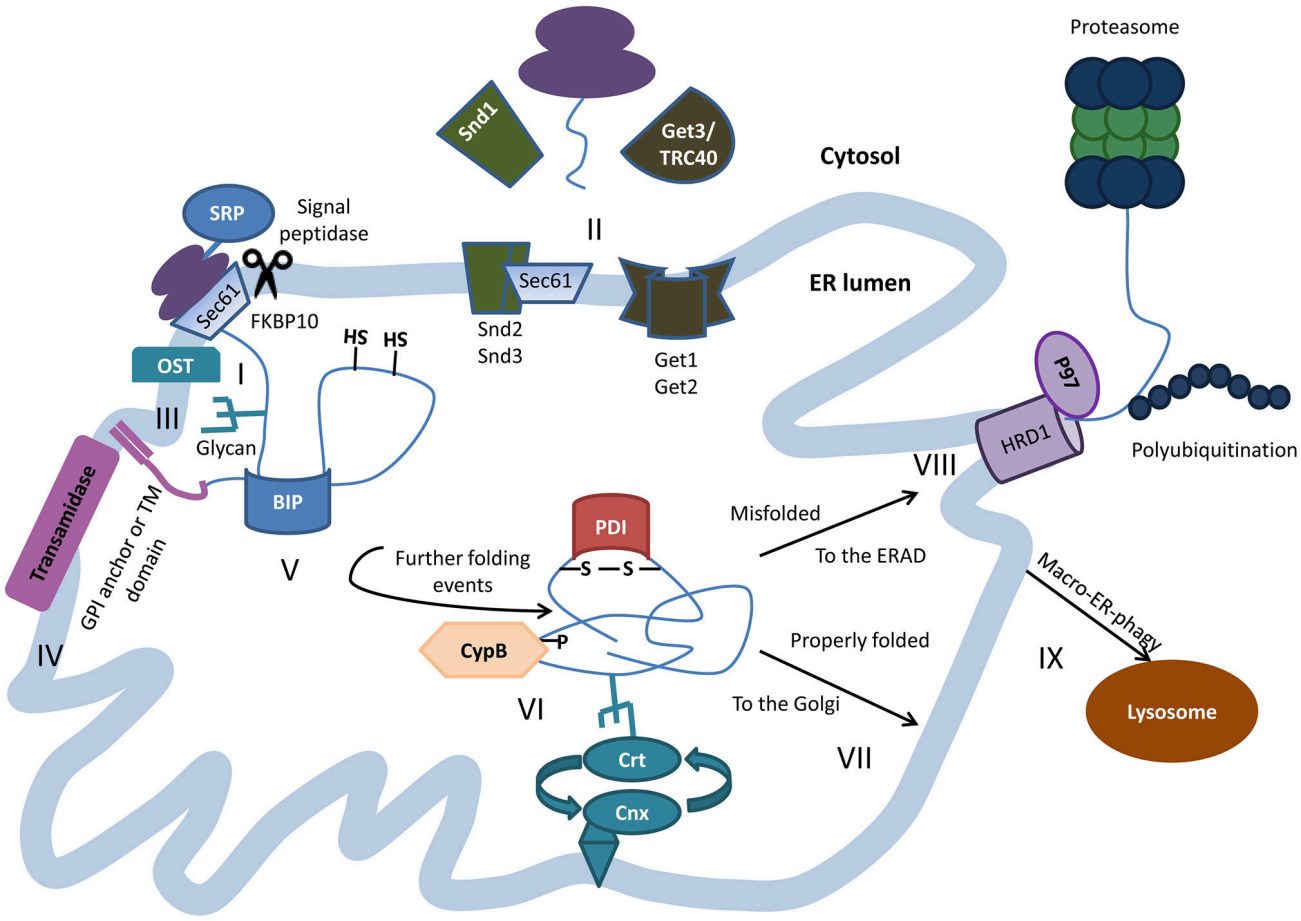
**Protein folding within the ER lumen**. Newly synthesized polypeptides are co-translationally translocated into the ER through the translocon Sec61p complex. Targeting and insertion of the signal peptide is mediated by signal recognition particle (SRP) and at least in the case of PrP, is assisted by FKBP10 (I). In some cases post translational insertion of secretory proteins occurs in an SRP-independent manner, as in case of the GET-mediated insertion of tail-anchored proteins or by the SND mechanism in other cases (II). As the nascent polypeptide exits the translocon, the signal peptide is cleaved and N-linked glycans are added co-translationally by oligosaccharyltransferase (OST) (III). Transmembrane proteins are inserted into the ER membrane and some proteins get anchored to the membrane through the addition of glycophosphatidylinositol (GPI) lipid tail by the transamidase complex (IV). The highly conserved chaperone BiP binds nascent polypeptides as they are translocated into the ER, and maintains them in a state competent for subsequent folding and oligomerization. (V). The peptide undergoes further processing with the help of additional chaperones and folding enzymes, among them the calnexin/calreticulin lectin chaperones, protein disulfide isomerase (PDI) that oxidizes cysteine residues to induce disulfide-bond formation, and the ER resident cyclophilin B (CypB) that catalyzes cis/trans isomerization on the axis of certain proline residues (VI). Properly folded proteins are shipped for further processing in the Golgi (VII). Terminally misfolded proteins are retro-translocated to the cytosol to be degraded by the proteasomes through the ER-associated protein degradation (ERAD), often mediated by the E3 ubiquitin ligase HRD1 and the ATPase VCP/p97 (VIII). Sometimes, ERAD substrates and excess membranes and membrane proteins are shuttles for degradation in the lysosome via macro-ER-phagy (IX).

Upon entry into the ER, the ER-localization signal is cleaved (Haeuptle et al., 1989), and many of the newly synthesized polypeptides are anchored to the ER membrane through their transmembrane domains. Some other proteins, including the prion protein (PrP), acquire a glycophosphatidylinositol (GPI) lipid tail (Muñiz and Zurzolo, 2014). Oligosaccharides are attached to asparagine residues of many newly synthesized proteins. These modifications render the polypeptides recognizable by lectin folding chaperones which play key roles in the folding and maturation of glycosylated proteins (Noack et al., 2014). These chaperones and folding-assisting enzymes (foldases) catalyze the molecule's maturation by a series of sequential events that help it attain its desired spatial structure (Figure 1). Examples of some well-characterized ER foldases are Calnexin (CNX), Calreticulin (CRT), the protein disulfide isomerase (PDI) ERp57 (Oliver et al., 1999), and cyclophilin B (CypB) (Jansen et al., 2012; for a comprehensive overview on ER-resident chaperones see (Gidalevitz et al., 2013). The interactions of the nascent polypeptide with CNX, CRT, and additional chaperones such as the Hsp70 family member BiP/GRP78 (Haas and Wabl, 1983), initiate the folding process and can recruit ERp57 (Kozlov et al., 2006) that catalyzes the formation of disulfide bonds. CypB, a member of the peptidylprolyl cis/trans isomerases (PPIases) family of chaperones, which utilizes specific proline residues to convert the maturating polypeptide from *cis* to *trans* position (Barik, 2006), emerges as a pivotal coordinator of the folding process (Jansen et al., 2012). FKBP10, a member of the FK506-binding proteins (FKBPs), an additional subgroup of PPIase chaperones, is needed to mediate the entry of certain nascent polypeptides into the ER (Stocki et al., 2016).

Despite the assistance of the intricate nexus of expert folding chaperones, subsets of newly synthesized proteins fail to fold properly and expose hydrophobic domains that lead to the aggregation of the protein. These terminally misfolded molecules are recognized by specialized ER-chaperones which impede their shuttling to the Golgi (Ellgaard and Helenius, 2003), and promote their destruction by the ER-associated degradation (ERAD). This process is executed by a conserved set of ERAD components, including the membrane integral, ERAD E3 ubiquitin ligase HRD1 (Bordallo et al., 1998) and the ATPase VCP/p97 that mediate the retro-translocation of unfolded polypeptides to the cytosol (Ye et al., 2004), and confer their degradation by the proteasome (Ruggiano et al., 2014). It is important to note that macro-autophagy emerges as an additional mechanism that play roles in the degradation of ERAD substrates (Lipatova and Segev, 2015). Under regular conditions the orchestrated activities of protein folding, quality control, and degradation mechanisms maintain proper protein homeostasis (proteostasis; Balch et al., 2008) however, in the face of stress, mutations or aging, subsets of proteins that bear the propensity to misfold, escape the cellular surveillance system, and form aggregates within the ER.

## ER stress responses

The accumulation of protein aggregates within the crowded environment of the ER lumen impairs its functionality and has the potential to be hazardous to the cell. Thus, highly conserved cellular mechanisms that refold unfolded polypeptides, clear aggregates, and restore functional proteostasis have been developed. One such well-defined mechanism is the unfolded protein response (UPR^ER^), a signaling cascade which has at least four arms that have similar principles of activity. When specialized ER proteins sense an accumulation of misfolded proteins, a sequence of events activates the UPR^ER^ which initiates the migration of transcription factors to the nucleus. The ATF6(N) fragment is cleaved from ATF and enter the nucleus, XBP1 is activated by IRE1 and modulates gene expression and ATF4 is shuttled to the nucleus by the PERK downstream mechanism. These transcription factors elevate the expression of genes that encode for ER chaperones (such as BiP) to enhance folding capacity and reduce the expression of genes that encode proteins that require the assistance of ER chaperones to fold properly, aiming to lower the aggregation challenge within the ER lumen (reviewed in Walter and Ron, 2011).

**Figure 2:**
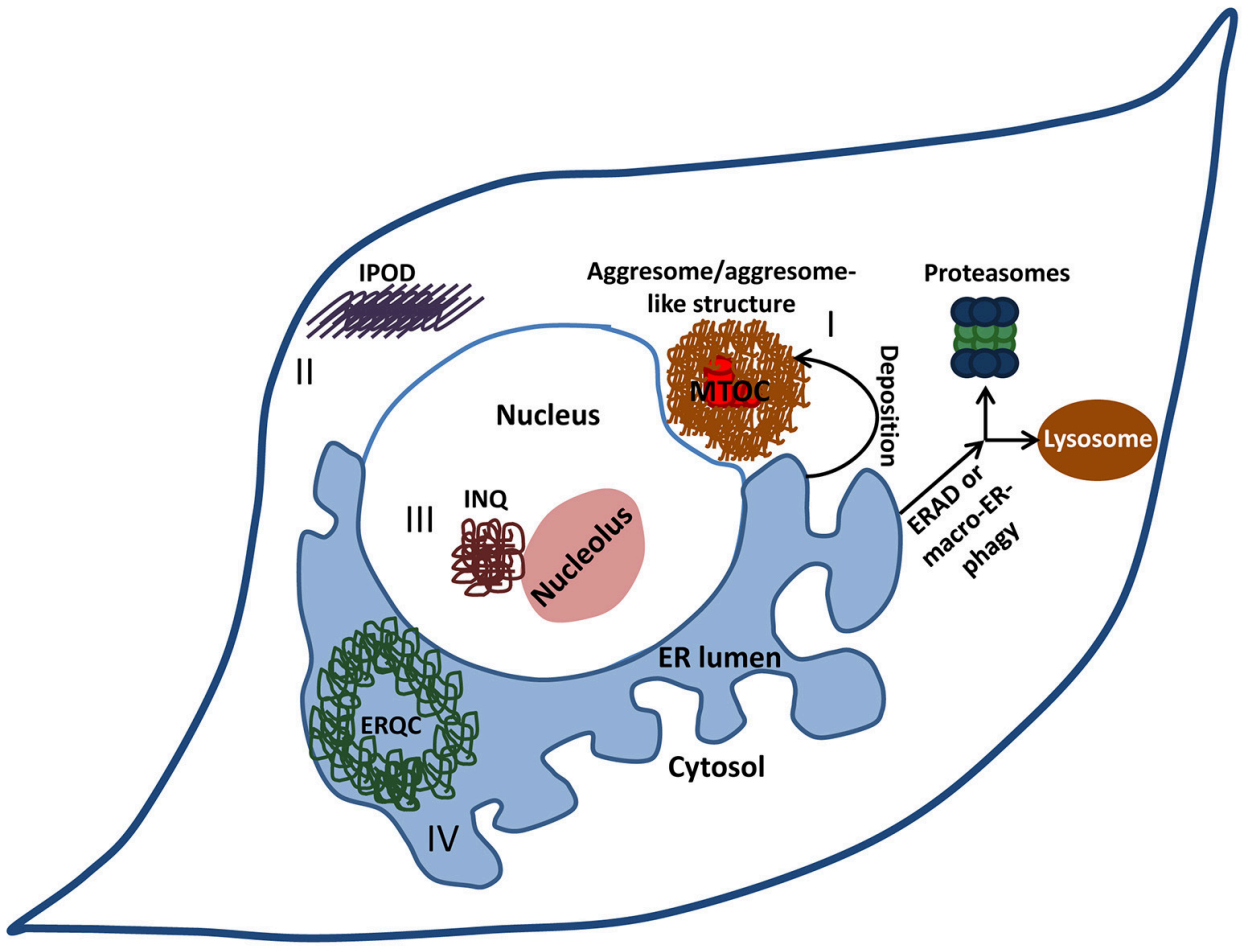
**Cellular deposition sites**. When the burden of misfolded proteins exceeds the cell's clearance and refolding capacity, potentially hazardous aggregates accumulate within the ER. Under certain circumstances, these aggregates are actively convoyed to designated deposition sites. Aggresomes or aggresome like-structures (I) are cytosolic juxta-nuclear inclusion bodies that serve as quality-control centers. Another type of a cytosolic deposition site is the insoluble protein deposit (IPOD) (II) where terminally aggregated proteins tend to accumulate. Intra-nuclear quality control compartment (INQ) (III) resides in the nucleus next to the nucleolus and harbors nuclear as well as cytosolic misfolded proteins. Some proteins that aggregate within the ER are deposited in the ER-derived quality-control compartment (ERQC) (IV).

Nevertheless, under certain circumstances, typically in late stages of life, the aggregation challenge exceeds the ER's protein folding and clearance capacities hindering the restoration of proteostasis. When potentially hazardous aggregates accumulate within the ER, they are actively convoyed to designated deposition sites (Figure 2). Such aggregated proteins are deposited next to the nucleus when proteasomes are inhibited. The formation of these cellular deposition foci was impeded by the inhibition of protein synthesis (Wójcik et al., 1996). These findings proposed that the juxta-nuclear, cellular foci are quality control compartments, where aggregates are temporarily stored to enable their degradation when conditions allow. A similar phenomenon of protein deposition in a cytosolic, nucleus-adjacent location was later reported. These sites, which were termed “aggresomes” (Johnston et al., 1998), contain aggregated membrane proteins and are positioned at the Micro Tubule Organizing Center (MTOC). We discovered that aggresomes which contain aggregated PrP are dynamic quality control compartments that attract molecular chaperones and proteasomes to mediate the degradation of their content (Ben-Gedalya et al., 2011). Additional types of cytosolic deposition sites where characterized in yeast and mammalian cells including the aggresomes-like, Juxta Nuclear Quality control compartment (JUNQ), and Insoluble Protein Deposit (IPOD) (Kaganovich et al., 2008). It is not entirely clear whether the JUNQ is cytosolic or nuclear, as a recent study claimed that the aggregates that were previously reported to accumulate in the cytosol are deposited within the nucleus (Miller et al., 2015). An additional type of deposition site is the “ER quality control compartment” (ERQC) which accumulates protein aggregates within the ER lumen (Kamhi-Nesher et al., 2001). Why certain protein aggregates accumulate in cytosolic sites while others are deposited within the ER is not known however, it is plausible that the cell fails to retro-translocate certain molecules to the cytosol and thus, deposits them in the ERQC.

In the face of aging-associated decline in protein quality control capabilities (Carvalhal Marques et al., 2015), or due to mutations that severely destabilize the three dimensional structures of aggregation-prone proteins, the cell fails to maintain proteostasis, and protein aggregates accumulate. Such uncontrolled protein aggregation can be toxic and underlie the development of proteinopathies (Paulson, 1999). Neurodegenerative maladies including Alzheimer's disease, Parkinson's disease (Selkoe, 2003), and prion disorders (Aguzzi and Calella, 2009) are a subgroup of proteinopathies. Late onset (Amaducci and Tesco, 1994) and the deposition of aggregated proteins in the brain are common features of these illnesses (Soto, 2003).

## Alzheimer's disease and prion disorders

Alzheimer's disease (AD), the most common type of dementia, is characterized by two pathological hallmarks; the deposition of small hydrophobic peptides known as β amyloid (Aβ) in plaques and the formation of Neurofibrillary Tangles (NFTs) of aggregated, hyper-phosphorylated microtubule-associated protein tau in the brain (Selkoe, 2011). According to the amyloid hypothesis (Hardy and Higgins, 1992), AD develops as a result of a dual cleavage of the Amyloid Precursor Protein (APP) by two proteases, the β and γ secretases. This digestion releases the family of Aβ peptides which form various types of oligomers and high molecular weight aggregates. Oligomers were found to be the most toxic Aβ species (Cohen et al., 2006; Shankar et al., 2008). The amyloid hypothesis proposes hyper Aβ production as a key mechanistic condition for the development of this devastating disorder (Hardy and Higgins, 1992). Similarly to other neurodegenerative disorders AD exhibits more than one pattern of occurrence. While most AD cases onset sporadically, individuals who carry mutations in the sequence of APP or of Presenilin 1 or 2 (both are components of the γ secretase complex) develop early-onset familial AD (fAD).

The misfolding and aggregation of the prion protein (PrP) underlies the development of several conformational diseases. Creutzfeldt-Jakob disease (CJD) is a fatal prion disorder that can onset sporadically, as a familial, mutation-linked malady as well as an infectious disease. It is well-documented that individuals who consumed contaminated beef developed CJD (Prusiner, 1998). Similarly to the case of Aβ, small oligomeric PrP structures are the most infectious prion species (Silveira et al., 2005). Interestingly, different mutations in the sequence of PrP lead to the development of distinct disorders. While certain mutations are accountable for the development of CJD, others underlie the manifestation of Gerstmann-Straussler-Scheinker disease (GSS; Salmona et al., 2003) or of Fatal Familial Insomnia (FFI; Medori et al., 1992). Unlike CJD, GSS, and FFI solely emerge as mutation-linked, familial disorders and exhibit much slower etiology.

## Failure of early maturation events underlie the onset of certain cases of neurodegeneration

Recent studies challenge the amyloid hypothesis suggesting that at least some fAD cases emanate from the attenuation of γ secretase activity that is inflicted by mutations in the sequence of presenilin 1. Analysis of γ secretase activity in brain samples of individuals who carried various fAD-associated mutations or developed sporadic AD (sAD) unveiled that in most cases the total levels of Aβ were lower than those observed in brains of individuals who showed no signs of dementia. Unexpectedly, no significant difference in total Aβ levels was detected among brains of individuals who had sAD and those of unaffected people (Szaruga et al., 2015). Another interesting avenue of research unveiled that in some cases, the loss of γ secretase endopeptidase function is associated with fAD. For instance, transgenic mice that harbor two copies of mutated presenilin 1 carrying either the L435F or C410Y fAD-linked mutations, exhibit near complete loss of γ secretase function but develop neurodegeneration (Xia et al., 2015). These findings clearly show that increased Aβ production is not a prerequisite for AD development and raise the question of what the mechanisms that underlie fAD are. Why these and other mutations lead to the loss of the γ secretase proteolytic function and whether protein maturation within the secretory pathway plays any role in the pathogenic process that leads to the development of fAD are largely unanswered questions.

To identify common mechanisms that initiate the development of neurodegenerative maladies, we searched for similar mutational patterns in different proteins which cause distinct neurodegenerative illnesses. This approach is based on the rationale that since folding chaperones of the secretion pathway assist the maturation process of many nascent polypeptides, it is probable that analogous mutations which impede chaperone-client interactions can lead to the development of distinct maladies. We identified similar PXXP motifs in the sequences of PrP and of presenilin 1. Previous reports indicated that the substitution of either proline in these motifs of PrP (Hsiao et al., 1989; Yamazaki et al., 1999) and presenilin 1 (Campion et al., 1995) cause GSS or fAD, respectively. To explore why the substitution of these prolines leads to misfolding and disease we used cultured cells and the cyclophilin-specific inhibitor cyclosporin-A (CsA) and found that when CypB is prevented from assisting presenilin 1 to fold properly, the nascent polypeptide misfolds, and forms aggregates that accumulate in the ERQC. This leads to severe impairment of γ secretase activity and to aberrant mitochondrial distribution and function. Similarly, the expression of presenilin 1 molecules carrying the fAD-linked mutations, P264L or P267L/S, resulted in the same phenotypes (Ben-Gedalya et al., 2015).

A similar mechanism triggers the pathogenic process that causes GSS. The inhibition of CypB by CsA or by the substitution of proline 102 or of 105 in the sequence of PrP resulted in the protein's aggregation and deposition in aggresomes (Cohen and Taraboulos, 2003).

Our studies show that hindering the interaction of CypB, a key ER-resident folding chaperone, with PrP and presenilin 1, results in the manifestation of certain cases of fAD or GSS. It is important to note that additional studies illustrate failures in other stages of protein maturation as the source of neurodegenerative disorders. The levels of CNX and CRT were found to be reduced in Parkinson's disease cellular model (Kuang et al., 2014) and ER stress response was found to be activated in amyotrophic lateral sclerosis (ALS)-model mice that express an ALS-linked mutated SOD 1 in their muscles (Chen et al., 2015). In addition, one of the early modifications that PrP undergoes upon entry to the ER is the attachment of GPI. Individuals who carry mutations, such as Q227Stop, that prevent GPI attachment to the newly synthesized molecule, express anchorless PrP and develop GSS (Jansen et al., 2010). It is plausible that anchorless PrP molecules cannot be recognized by ER chaperones and thus form aggregates within this organelle. This possibility may be supported by the report that similarly to CypB, CNX interacts with PrP and reduces PrP-mediated toxicity (Wang et al., 2010). Nevertheless, further research is needed to examine this hypothesis.

The accumulation in aggresomes of molecules that failed to fold properly within the ER, raises the question of whether these deposition sites (Ben-Gedalya et al., 2011) are cytosolic components of an ER-resident protein quality control mechanism or whether they also serve other cellular organelles as aggregate disposal centers.

## Aggresomes are cytosolic components of the ER protein quality control mechanism

The association of aggresomes with neurodegenerative maladies was first demonstrated by the accumulation of the fAD-causing, presenilin-1 which carries the A246E mutation in these structures (Johnston et al., 1998). Toxic PrP species (Kristiansen et al., 2005), disease causing PrP mutants (Cohen and Taraboulos, 2003; Mishra et al., 2003), and Parkinson's disease-associated, aggregated α-synuclein (Tanaka et al., 2004; Wong et al., 2008) were also shown to be deposited in aggresomes of mammalian cells, further linking these sites with a myriad of human neurodegenerative illnesses. To test whether aggresomes are mechanistically linked to the ER quality control machinery we created fluorescently-tagged PrP constructs that either efficiently enter the ER or stay entirely cytosolic and tested whether these molecules are deposited in aggresomes upon exposing the cell to CsA treatment. We found that PrP must enter the ER in order to be deposited in the aggresome (Dubnikov et al., 2016) defining these sites as remote cytosolic components of the ER. Interestingly, the attachment of a GPI anchor is needed for the direction of PrP to the aggresome but the Golgi apparatus appears to have no role in shuttling aggregated PrP to this structure. This insight further associates the ER protein folding mechanism with deposition sites, a key hallmark of neurodegenerative diseases, and shows that proper activity of folding chaperones protects from these disorders. However, is foldase activity always protective?

Recent studies have shown that counter-intuitively, knocking down the activity of certain chaperones protects from neurodegeneration. The PPIase FKBP51 acts in collaboration with Hsp90 to prevent the clearance of aggregated tau enhancing the protein's oligomerization. Accordingly, mice over-expressing FKBP51 suffered from neurotoxicity (Blair et al., 2013). Recently, it was shown that reducing the rate of PrP entry into the ER by the inhibition of the ER-resident PPIase FKBP10, reduces toxicity in mammalian cell systems (Stocki et al., 2016), suggesting that under these specific circumstances this foldase enhances PrP toxicity. It is possible that FKBP51 and FKBP10 enhance proteotoxicity not by the modulation of protein folding but by exhibiting other functions and that the activities of certain chaperones may be protective in the face of certain proteotoxic challenges but deleterious in the face of others.

## Therapeutic opportunities

Accumulating information point at the ER lumen as an important arena where neurodegenerative-causing events occur. Accordingly, interventions that modulate the activity of ER components can help cells maintain functional proteostasis, prevent the accumulation of hazardous aggregates within the lumen and delay the manifestation of neurodegenerative maladies. Nevertheless, the emerging understanding that various mechanisms can underlie the development of the same malady and the apparent damaging functions of certain folding chaperones requires a careful characterization and classification of neurodegeneration-causing mechanisms. The approval of proteostasis-enhancing compounds and their high efficacy for the treatment of Cystic Fibrosis (Van Goor et al., 2009; Carter et al., 2015) and the counter-proteotoxic effects of aging-modulating compounds (El-Ami et al., 2014) are very encouraging developments which indicate that a comprehensive understanding of the mechanisms that underlie proteinopathies is the basis for the development of novel therapies for hitherto incurable, devastating disorders.

## Author contributions

EC and TD wrote the manuscript. TD prepared the figures.

## Funding

Our studies that are described in this manuscript were generously supported by the European Research Council (ERC, #281010) and the Israel Science Foundation (ISF #981/16).

### Conflict of interest statement

The authors declare that the research was conducted in the absence of any commercial or financial relationships that could be construed as a potential conflict of interest.
